# Identification of Hub Biomarkers and Immune and Inflammation Pathways Contributing to Kawasaki Disease Progression with RT-qPCR Verification

**DOI:** 10.1155/2023/1774260

**Published:** 2023-04-06

**Authors:** Hongjun Ba, Lili Zhang, Huimin Peng, Xiufang He, Yuese Lin, Xuandi Li, Shujuan Li, Ling Zhu, Youzhen Qin, Xing Zhang, Yao Wang

**Affiliations:** ^1^Department of Pediatric Cardiology, Heart Center, First Affiliated Hospital of Sun Yat-sen University, 58# Zhongshan Road 2, Guangzhou 510080, China; ^2^Key Laboratory on Assisted Circulation, Ministry of Health, 58# Zhongshan Road 2, Guangzhou 510080, China; ^3^Department of Cardiology, Kunming Children's Hospital, 288 Qianxing Road, Xishan District, Kunming 650034, Yunnan, China; ^4^Cancer Hospital, Guangzhou Medical University, Guangzhou 510095, China

## Abstract

**Background:**

Kawasaki disease (KD) is characterized by a disordered inflammation response of unknown etiology. Immune cells are closely associated with its onset, although the immune-related genes' expression and possibly involved immune regulatory mechanisms are little known. This study aims to identify KD-implicated significant immune- and inflammation-related biomarkers and pathways and their association with immune cell infiltration. *Patients and Methods*. Gene microarray data were collected from the Gene Expression Omnibus database. Differential expression analysis, weighted gene coexpression network analysis (WGCNA), least absolute shrinkage and selection operator (LASSO) regression, Gene Ontology (GO), Kyoto Encyclopedia of Genes and Genomes (KEGG), and gene set enrichment analysis (GSEA) were used to find KD hub markers. GSEA was used to assess the infiltration by 28 immune cell types and their connections to essential gene markers. Receiver operating characteristic (ROC) curves were used to examine hub markers' diagnostic effectiveness. Finally, hub genes' expressions were validated in Chinese KD patients by reverse transcription-quantitative polymerase chain reaction (RT-qPCR).

**Results:**

One hundred and fifty-one unique genes were found. Among 10 coexpression modules at WGCNA, one hub module exhibited the strongest association with KD. Thirty-six overlapping genes were identified. Six hub genes were potential biomarkers according to LASSO analysis. Immune infiltration revealed connections among activated and effector memory CD4^+^ T cells, neutrophils, activated dendritic cells, and macrophages. The six hub genes' diagnostic value was shown by ROC curve analysis. Hub genes were enriched in immunological and inflammatory pathways. RT-qPCR verification results of *FCGR1B* (*P* < 0.001), *GPR84* (*P* < 0.001), *KREMEN1* (*P* < 0.001), *LRG1* (*P* < 0.001), and *TDRD9* (*P* < 0.001) upregulated expression in Chinese KD patients are consistent with our database analysis.

**Conclusion:**

Neutrophils, macrophages, and activated dendritic cells are strongly linked to KD pathophysiology. Through immune-related signaling pathways, hub genes such as *FCGR1B*, *GPR84*, *KREMEN1*, *LRG1*, and *TDRD9* may be implicated in KD advancement.

## 1. Introduction

Kawasaki disease (KD), often called cutaneous lymph node syndrome, was initially described by Tomisaku Kawasaki in 1967 [1]. Children under 5 years of age are primarily affected by KD. KD is an acute systemic immune vasculitis caused by infectious factors that can be complicated by coronary artery lesions (CAL). CAL caused by KD are a common heart disease in some countries and regions. On the other hand, the steps leading to the development of KD are not fully understood. The diagnosis of KD depends on typical clinical manifestations. However, some children with KD have atypical manifestations and are easily misdiagnosed with other diseases, leading to an increased risk of coronary artery damage due to delayed diagnosis and treatment [2, 3]. Therefore, identifying a biomarker to diagnose KD helps with early diagnosis and treatment, reducing the risk of damage to the coronary arteries.

Although much research has been conducted, the pathogenesis of KD is not completely known. Recent research has suggested that immune cells may play a role in KD development and its associated symptoms. Alterations in the monocyte development locus can be observed during the acute phase of KD infection [4]. When KD is in its acute phase, CD8^+^ T cell expression decreases significantly [5]. Immunohistochemical analysis of coronary arteries of deceased patients with KD showed the presence of monocytes, macrophages, neutrophils, monocytes, macrophages, and activated CD8^+^ T cells [6] and IgA^+^ plasma cells [7, 8] in the arterial wall. Furthermore, immune regulatory genes such as *CXCL8* and *CCL5*, among others, have the potential to play a crucial role in the progression of KD [9]. These findings imply that genes related to the immune system may be involved in the pathogenesis of KD.

Bioinformatic analysis approaches include least absolute shrinkage and selection operator (LASSO) and weighted gene coexpression network analysis (WGCNA). WGCNA aggregates similar-expressed genes into a single module using clustering. This approach has substantial biological consequences and can effectively screen for target-related genes [10, 11]. Unlike traditional Cox and logistic regression methods, the LASSO regression method aims to gain insight into the exact degree of connection between two inextricably connected variables. The accuracy of character-related gene screening may be improved by LASSO analysis of WGCNA genes [12]. The WGCNA and LASSO technologies were used to identify important KD biomarkers. Using DEGs, or differentially expressed genes, these biomarkers were identified. The immune-related signaling pathways linked with DEGs were then identified using Gene Ontology (GO), the Kyoto Encyclopedia of Genes and Genomes (KEGG), and gene set enrichment analysis (GSEA). This study is the first to evaluate 28 immune cell infiltrates using a single GSEA sample (ssGSEA) to better understand the pathogenesis of KD and treatment goals. Finally, we validated the screening of hub genes in Chinese KD patients.

## 2. Materials and Methods

### 2.1. Data Extraction

The Gene Expression Omnibus database (https://www.ncbi.nlm.nih.gov/geo/) was used to get microarray expression data and clinical information relevant to KD (GSE18606 [13], GSE73461 [14], and GSE68004 [15]). In the GSE18606 dataset, 47 samples were taken from 38 patients diagnosed with KD, and nine healthy individuals served as controls. Within the GSE73461 dataset, there were 78 KD and 55 control samples. The GSE68004 collection included 113 samples: 76 patients with KD and 37 healthy controls. GSE18606 and GSE73461 were added to the metadata queues (training groups) in preparation for future integration investigations. The training group was validated using the GSE68004 dataset as the test group.

### 2.2. Identification of DEGs

For data normalization and probe annotation, the “limma” and “GEOquery” packages included in R software (version 4.0.1) were applied. The screening criterion for DEGs consisted of an adjusted *P*-value of <0.05 and a log fold change (FC) of more than 1 [16, 17].

### 2.3. Construction of Gene Coexpression Network

The WGCNA package of the R program was used to build a weighted coexpression network from the expression profile data of the training group (in addition to GSE18606 and GSE73461), and 25% of the genes that were the most distinct from the median were selected for the investigation [10]. The “goodSampleGenes” function was used to validate the accuracy of the data. The “pickSoftThreshold” function was used to calculate and confirm that the optimal soft threshold (*ß*) had been attained. Clustering was performed to identify the modules most similar in terms of their topological overlap after the matrix data were converted into an adjacency matrix. After the module characteristic genes of the modules had been calculated, a hierarchical clustering tree diagram was built according to the comparable modules that were present in the cluster tree of the feature genes. This investigation integrated phenotypic and modular data to determine gene significance (GS) and modular importance. Furthermore, a determination was made about the value of genetic and clinical information, and research was carried out on the correlation between modules and models. The method was determined to compute the degree of each gene's module membership (MM), and the GS values of each module were analyzed. The adjacency matrix was constructed by a weighted correlation coefficient. Subsequently, the adjacency matrix was transformed into a topological overlap matrix (TOM). Then, hierarchical clustering was performed to identify modules, and the eigengene was calculated. Finally, we assessed the correlation between phenotype (KD or control samples) and each module by Pearson's correlation analysis and identified KD-related modules. The genes in these modules were considered KD-related module genes.

### 2.4. Screening and Validation of Hub Genes

To use them as possible hub genes, priority was given to genes exhibiting the highest level of intermodular connection. Generally, genes that are more important biologically have more significant absolute amount of GS. The default was used as the basis for the candidate gene screening criteria of GS absolute value >0.50 and MM absolute value >0.80. It was determined that the genes with the highest intermodular connection levels should be prioritized for use as potential hub genes. In general, genes with greater biological relevance have higher levels of absolute GS.

### 2.5. Immune Cell Infiltration and Its Association with Hub Genes

In the training group, the relative amounts of infiltration of each of the 28 different types of immune cells were measured using the ssGSEA technique [18]. The varying degrees of expression of each of the 28 different types of immune cells are shown as a violin diagram. The “ggplot2” software application was used to display the results of a calculation that determined the Spearman correlation between the hub gene and the 28 immune cells.

### 2.6. Functional Enrichment Analysis

The R packages “clusterProfiler” and “enrichment plot” were utilized to study DEGs according to GO, KEGG, and GSEA. GSEA analysis was performed based on all genes. The level of statistical significance was obtained when *P* was <0.05 [19]. The ssGSEA method was used to determine the relative amounts of infiltration of 28 different types of immune cells in the training group [18]. It was shown using a violin diagram how the different types of immune cells each had a distinct amount of differential expression. The “cor.test” function was utilized to calculate the Spearman correlation between the hub gene and the 28 immune cells. ggplot2 software application was used for visualization.

### 2.7. Validation of Hub Genes

Reverse transcription-quantitative polymerase chain reaction (RT-qPCR) assays were performed to verify the reliability of bioinformatics-based results. A total of 20 study participants were recruited from the First Affiliated Hospital of Sun Yat-sen University, including 10 KD patients and 10 healthy controls. All subjects gave written informed consent in accordance with the Declaration of Helsinki. The protocol was approved by the Ethics Committee of the First Affiliated Hospital of Sun Yat-sen University ([2022]514). Peripheral venous blood was collected from each participant; then, total RNA was extracted from each sample using TRIzol Reagent (Invitrogen, United States) according to the manufacturer's instructions. The cDNA was synthesized using the SuperScript III Reverse Transcriptase Kit (Invitrogen, United States). RT-qPCR was performed with Power SYBR Green PCR Master Mix (TransGen Biotech, China) on an ABI 7,500 fast real-time PCR system. The amplification reaction procedure was as follows: 95°C for 10 min, followed by 95°C for 15 s and 60°C for 1 min for 40 cycles [20]. GAPDH was selected as the internal control for mRNA, and the relative expression level of mRNA was calculated by the relative quantification (2^−*ΔΔ*Ct^) method. Primer sequences are listed in *Supplementary 1*.

## 3. Results

**Figure 1:**
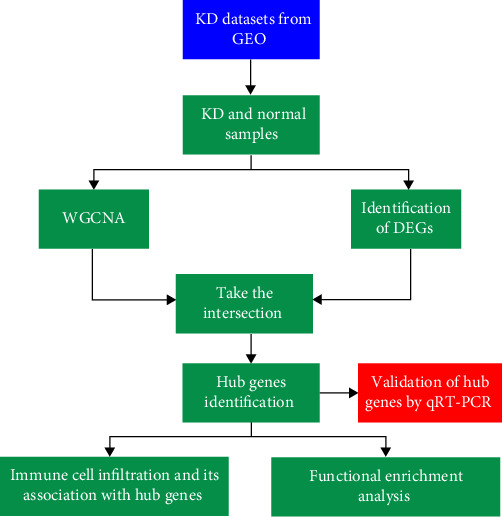
Flowchart of the research process.

The study's flowchart is shown in Figure 1.

### 3.1. Coexpression Network Construction and Identification of Hub Modules

**Figure 2:**
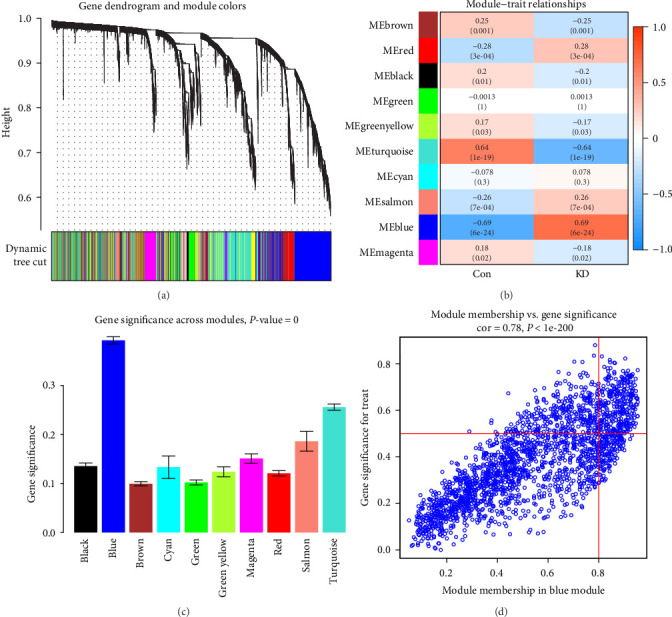
Development of the creation of WGCNA modules: (a) cluster dendrogram of genes whose median absolute deviation is among the top 25% of the range. The lines in the diagram represent a gene, and the colors denote the coexpression modules associated with those genes; (b) a heatmap illustrates the relationships between the modules and the traits. A powerful connection was found between the blue module and KD; (c) a representation of the distribution of the mean gene significance between KD-related modules; (d) a scatter plot shows the connection between membership in the blue module of the gene module and the level of relevance of the gene. Abbreviations: KD, Kawasaki disease; WGCNA, weighted gene coexpression network analysis.

The data from 10,869 genes in the top 25% of absolute departure from the median were pooled to produce the WGCNA. An identification procedure for missing values was carried out, and outliers were removed. The value of *ß* = 3 was determined to be the soft threshold (scale-free *R*^2^ = 0.85, slope = −1.21), which agreed with the scale-free network (*Supplementary 2 and 3*). A coexpression matrix was constructed using a one-step method, and 10 gene modules were obtained using the dynamic mixed shear method (Figure 2(a)). The correlation between the above modules and KD and healthy controls was displayed using heat maps. A hub module (blue module, comprising 315 genes) had the strongest connection with KD (cor = 0.69; *P* = 6e-24) (Figures 2(b) and 2(c)). In addition, there was a strong correlation between GS and MM inside the blue module (cor = 0.78; *P* = 1e-200), suggesting that the two may be connected (Figure 2(d)). In addition, the blue module served as the primary focus for the subsequent analyses.

### 3.2. Identification of DEGs and Screening of Hub Genes

**Figure 3:**
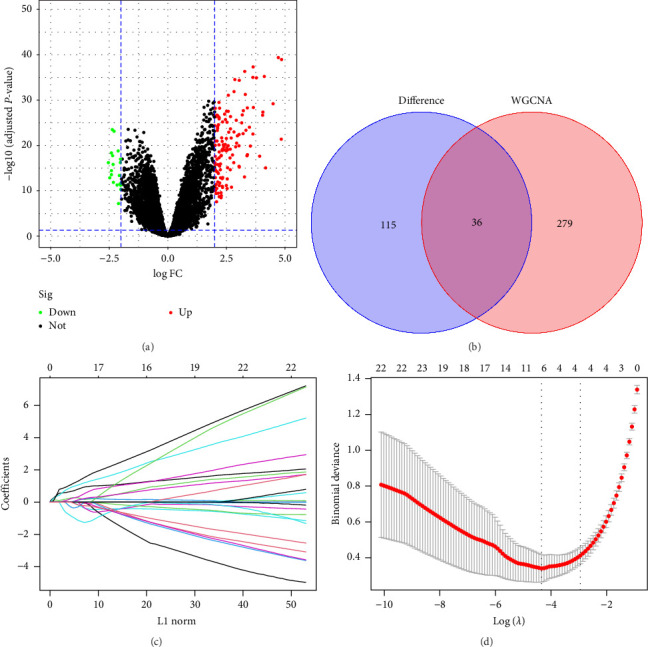
Detection of differentially expressed genes and selection of hub genes: (a) a volcano plot shows DEGs that compare healthy control tissues with those affected by KD; (b) a Venn diagram depicting the intersection of DEGs and the blue module. Thirty-six DEGs were identified as intersecting; (c) comparing LASSO regression with partial likelihood deviance while altering log (l) in 10-fold cross-validations. Vertical dashed lines mark the ideal (lambda.min) values defined by the minimal criterion (1-SE criterion); (d) LASSO coefficient profiles were produced from 10-fold cross-validation for the six hub genes. Abbreviations: DEGs, differentially expressed genes; KD, Kawasaki disease; LASSO, least absolute shrinkage and selection operator.

After correcting the *P*-values to <0.05 and |logFC| to >1, a total of 151 DEGs were identified, 132 and 19 of which had an increase and a decrease in expression, respectively. These DEGs were discovered using a volcano plot (Figure 3(a)). The applicant completed the screening process since their absolute GS score was >0.50 and their absolute MM score was >0.80. Based on these overlaps, 36 DEGs were identified as intersecting (Figure 3(b)). Six hub genes, namely, Fc gamma RI (*FCGR1*), G protein-coupled medium-chain fatty acid receptor (*GPR84*), haptoglobin (*HP*), kringle-containing transmembrane protein 1 (*KREMEN1*), leucine-rich *α*-2-glycoprotein 1 (*LRG1*), and tudor domain-containing protein 9 (*TDRD9*), were identified by LASSO analysis (Figures 3(c) and 3(d)).

### 3.3. Functional Enrichment Analysis of DEGs

**Figure 4:**
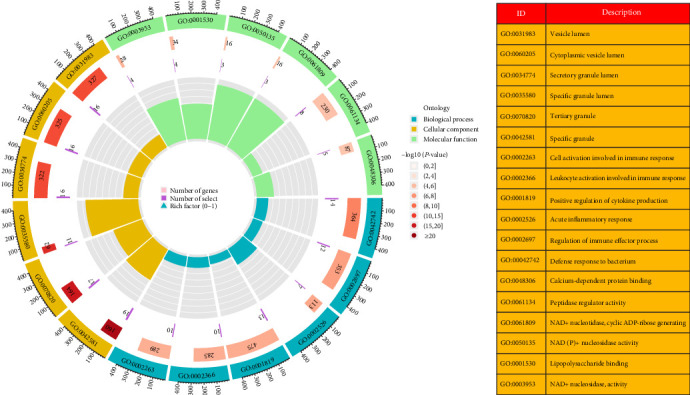
Using GO keywords in the study of differentially expressed genes in biological processes, the first lap represents the top 18 GO keywords, while the outer lap indicates the total number of genes. During the second pass, the number of genes already present in the genomic background and the *P*-values linked with the enrichment of DEGs for biological processes were retrieved. In the third lap, we compare the number of genes that were elevated (dark purple) with the number of genes that were downregulated (light purple). The fourth circuit represents the enrichment factor of each GO word in the diagram in the figure. Abbreviations: DEG, differentially expressed genes; GO, Gene Ontology.

**Figure 5:**
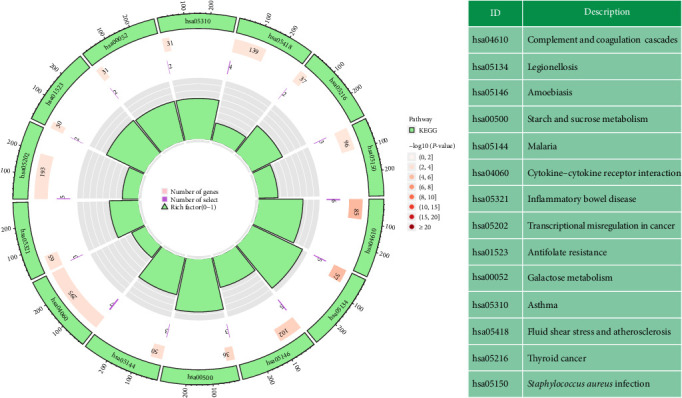
Utilizing terminology from the KEGG database to research DEGs in metabolic processes, organismal systems, and human diseases. The first lap depicts the top 14 KEGG keywords, while the outer lap represents the total number of genes in the human genome. In the second lap, the *P*-values for DEG enrichment are displayed along with the total number of genes present in the genetic background. In the third lap, we compare the number of genes that were elevated (dark purple) with the number of genes that were downregulated (light purple). The enrichment factor for each KEGG term is presented in the fourth circuit of the scheme. Abbreviations: DEGs, differentially expressed genes; KEGG, Kyoto Encyclopedia of Genes and Genomes.

We found the biological activities and signaling pathways linked with KD-related DEGs by researching the GO and KEGG pathways. According to the findings of the GO enrichment study, DEGs were much more prevalent in immunological and inflammatory processes (such as activation of cells, including white blood cells, involved in immune responses, positive regulation of cytokine production, and acute inflammatory reactions) (Figure 4). Based on an analysis of the KEGG signaling network, DEGs enriched immune and inflammatory disorders (e.g., inflammatory bowel disease and asthma), immune-related pathways (e.g., cytokine–cytokine receptor interaction), and infectious diseases (e.g., inflammatory bowel disease and asthma) (Figure 5). These results showed that abnormal signaling pathways and cellular processes cause the progression of KD.

### 3.4. Identification of Hub Gene Expression Levels and Diagnostic Value

**Figure 6:**
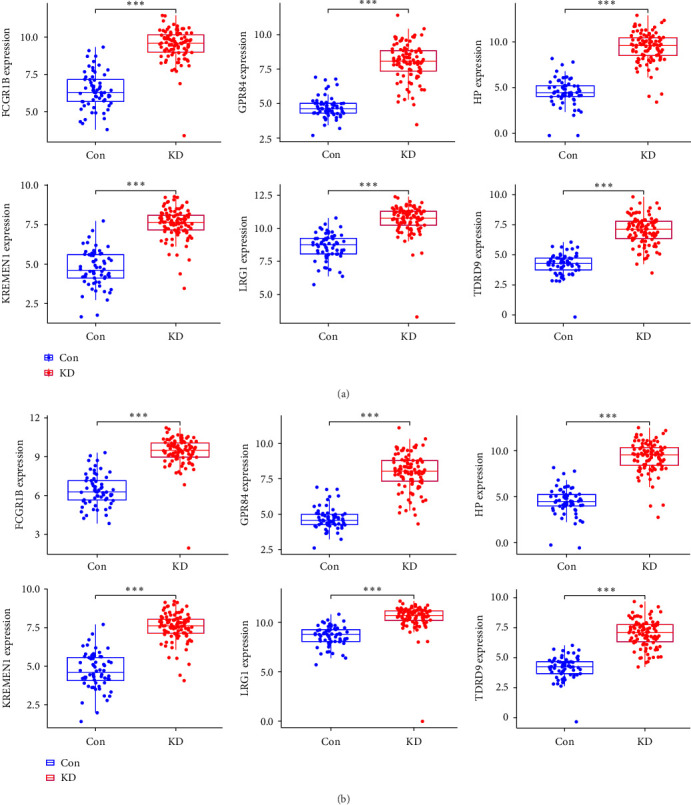
Hub genes' validation performed at the level of gene expression: (a) verification of the expression of hub genes in the training population (merging GSE18606 and GSE73461 datasets). KD patients had higher levels of *FCGR1B*, *GPR84*, *HP*, *KREMEN1*, *LRG1*, and *TDRD9* than healthy controls; (b) the validation of the hub genes in the test group (GSE68004) yielded results that overlapped with the results of the training group. Abbreviation: KD, Kawasaki disease. *⁣*^*∗∗∗*^*P* < 0.001.

As part of the verification process, box plots were utilized to examine the expression levels produced by six different hub genes. *FCGR1B* (*P* < 0.001), *GPR84* (*P* < 0.001), *HP* (*P* < 0.001), *KREMEN1* (*P* < 0.001), and *LRG1* (*P* < 0.001) all had significantly higher expression levels in KD tissues than in healthy control tissues (Figure 6(a)). Subsequently, the levels of expression of these six hub genes were examined using a second external dataset known as GSE68004, and it was demonstrated that they were accurate (Figure 6(b)).

**Figure 7:**
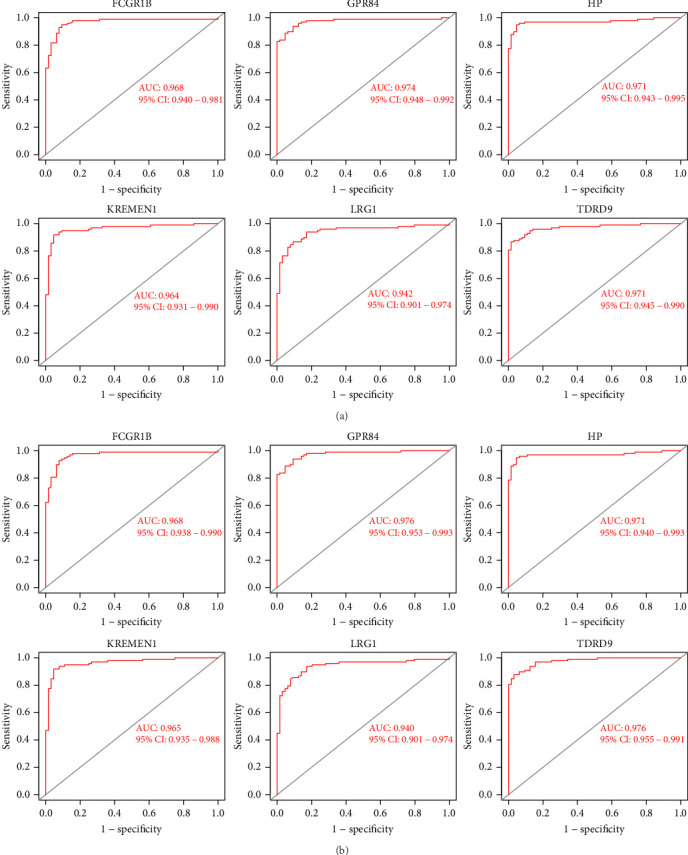
Validation of hub genes in the diagnostic value: (a) validation of diagnostically relevant hub genes (fusion of GSE18606 and GSE73461). ROC curves and area under the curve (AUC) data were utilized to examine the capability to distinguish KD from healthy controls with high sensitivity and specificity; (b) results from the validation of the hub genes in the test group (GSE68004) were comparable to those obtained in the training group. According to these data, the diagnostic sensitivity of these six hub genes for KD is at the higher end of the spectrum. Abbreviations: AUC, area under the curve; KD, Kawasaki disease; ROC, receiver operating characteristic.

An investigation of the receiver operating characteristic (ROC) curve was conducted to evaluate how sensitive or specific a diagnosis of KD is. As a result, the values of the area under the curve (AUC) for the six key genes could be compared. Because the AUC values for the six hub genes were higher than 0.94, it was clear that these genes have a solid diagnostic value for KD (Figure 7(a)). We conducted further tests to establish the diagnostic importance of the six hub genes in the GSE68004 dataset. AUC values >0.90 were observed for each of the six hub genes (Figure 7(b)).

### 3.5. Correlation between Immune Cell Infiltration and Hub Genes

**Figure 8:**
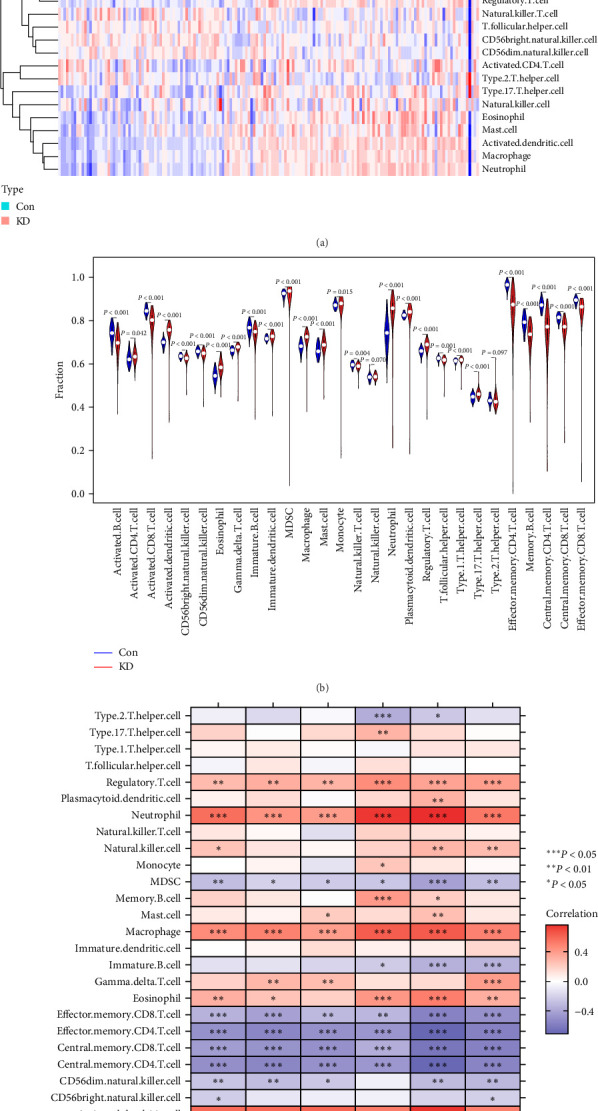
Examining the immune landscape associated with Kawasaki disease. A heat map (a) and a violin plot (b) graphically representing the distribution of the 28 different immune cells found in healthy control tissues and KD tissues, respectively. (c) the connection between immune cell infiltration and the activity of six hub genes found in the human genome. Abbreviation: KD, Kawasaki disease.

The ssGSEA approach was applied to evaluate the differences in immune cell infiltration between patients with KD and healthy controls. This was done to determine a relationship between KD and healthy controls.  Figure 8(a) shows the distribution of the 28 immune cells in the training group. It was discovered that tissues of KD patients have a significantly higher level of infiltration of activated and effector memory CD4^+^ T cells, monocytes, neutrophils, and macrophages than healthy tissues. This study was conducted on the topic of immune cell infiltration. This study supports the hypothesis that these cells are necessary to develop KD (Figure 8(b)).

According to the findings of an investigation into the connections between 28 distinct types of immune cells and hub genes, there are positive associations between neutrophils and *FCGR1B* (cor = 5.655; *P* < 0.001), *GPR84* (cor = 4.281; *P* < 0.001), *HP* (cor = 3.918; *P* < 0.001), *KREMEN1* (cor = 7.367; *P* < 0.001), *LRG1* (cor = 7.552; *P* < 0.001), and *TDRD9* (cor = 5.321; *P* < 0.001) (Figure 8(c)).

Positive correlations were found between activated dendritic cells (DCs) and the following genes: *FCGR1B* (cor = 5.807; *P* < 0.001), *GPR84* (cor = 5.861; *P* < 0.001), *HP* (cor = 5.995; *P* < 0.001), *KREMEN1* (cor = 5.169; *P* < 0.001), *LRG1* (cor = 7.448; *P* < 0.001), and *TDRD9* (cor = 5.503; *P* < 0.001). Positive correlations were found between macrophages and the following genes: *FCGR1B* (cor = 4.609; *P* < 0.001), *GPR84* (cor = 4.979; *P* < 0.001), *HP* (cor = 3.890; *P* < 0.001), *KREMEN1* (cor = 6.242; *P* < 0.001), *LRG1* (cor = 6.348; *P* < 0.001), and *TDRD9* (cor = 4.983; *P* < 0.001).

Negative correlations were found between effector memory CD4^+^ T cells and *FCGR1B* (cor = −4.995; *P* < 0.001), *GPR84* (cor = −5.485; *P* < 0.001), *HP* (cor = −4.987; *P* < 0.001), *KREMEN1* (cor = −4.732; *P* < 0.001), *LRG1* (cor = –7.058; *P* < 0.001), and *TDRD9* (cor = –5.765; *P* < 0.001).

Negative correlations were found between central memory CD4^+^ T cells and *FCGR1B* (cor = –4.399; *P* < 0.001), GPR84 (cor = –4.897; *P* < 0.001), *HP* (cor = –4.977; *P* < 0.001), *KREMEN1* (cor = –3.654; *P* < 0.001), *LRG1* (cor = –6.304; *P* < 0.001), and *TDRD9* (cor = –5.739; *P* < 0.001).

In addition, a negative connection was seen between the six hub genes and effector memory CD8^+^ T cells and central memory CD8^+^ T cells (Figure 8(c)). These findings support the hypothesis that specific immune cells play an essential role in the development of KD.

### 3.6. Enrichment Analysis of GSEA Immune Signature Gene Sets

**Figure 9:**
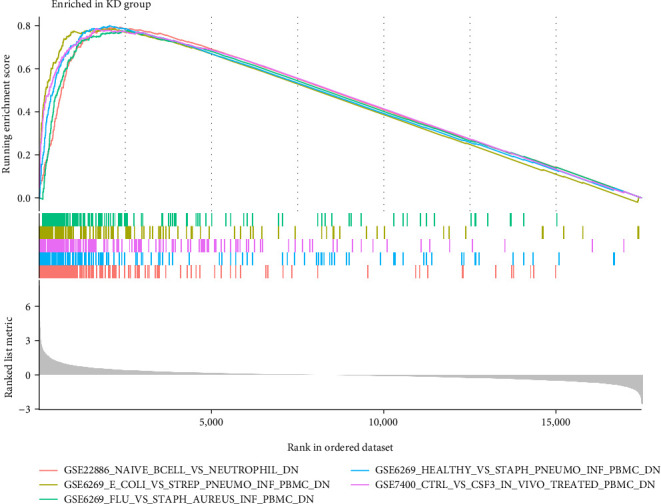
Enhancement diagram for the GSEA immunologic signature database.

To investigate the most likely mechanism that keeps immune function intact throughout the course of KD, we used the Molecular Signatures Database (MsigDB) of immunological signature genes as a reference for GSEA of DEGs. This analysis focused on genes that were expressed at different levels. Eight hundred and thirty-three gene sets showed substantially higher enrichment, as shown by a normalized enriched score (NES) >1 and a false discovery rate (FDR) *q*-value <0.05. It was shown that neutrophils and peripheral blood mononuclear cells (PBMCs) have much more significant enrichment in these genes. The 15 gene sets that are the most enriched are given in Table 1. The findings of this investigation support the hypothesis that immune-related genes play a significant role in the onset of KD and its progression (Figure 9).

### 3.7. RT-qPCR Validation of Hub Genes in Chinese KD Patients

**Figure 10:**
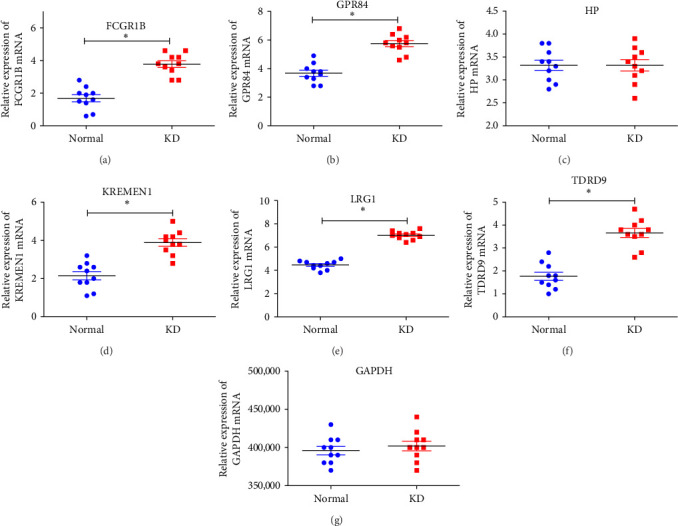
RT-qPCR validation of hub genes in KD patients: (a) expression level of *FCGR1B* gene; (b) expression level of *GPR84* gene; (c) expression level of *HP* gene; (d) expression level of *KREMEN1* gene; (e) expression level of *LRG1* gene; (f) expression level of *TDRD9* gene; (g) expression level of *GAPDH* gene. *⁣*^*∗*^*P* < 0.001.

To further verify the expression of the 10 hub genes in KD patients, we detected their expression in 20 peripheral venous blood samples from 10 KD patients and 10 samples from healthy individuals. The results showed that the expression of *FCGR1B* (*P* < 0.001), *GPR84* (*P* < 0.001), *KREMEN1* (*P* < 0.001), *LRG1* (*P* < 0.001), and *TDRD9* (*P* < 0.001) was significantly upregulated in KD patients compared with the control group, while *HP* (*P* > 0.05) was not significantly upregulated in KD patients compared to the normal group (Figure 10 and *Supplementary 4*).

## 4. Discussion

In recent years, the widespread application of high-throughput microarray technology has resulted in developing more rapid and effective bioinformatics methods to screen critical genes associated with disease mechanisms, occurrence, and progression. These methods have been made possible because of the widespread application of high-throughput microarray technology. Because of this, effective ways of diagnosing diseases, designing new drugs, and treating existing patients have been developed. Our research revealed that immunological responses, positive regulation of cytokine production, and acute inflammatory responses were more prevalent in DEGs in patients with KD compared to healthy controls. Based on these findings, specific pathways were implicated in the development of KD [21, 22].

Previous research found that neutrophils, monocytes, and macrophages increased considerably in peripheral blood samples from individuals with acute KD [23]. Interleukin (IL)-6, a soluble receptor for tumor necrosis factor, rises considerably during the acute phase of KD [24, 25]. When KEGG signaling pathways were examined, DEGs were shown to be more abundant in immunological and inflammatory diseases (such as inflammatory bowel disease and asthma), immune-related pathways (such as cytokine–cytokine receptor interaction), and viral diseases (e.g., inflammatory bowel disease and asthma). These results provide additional credence to the hypothesis that the development of KD is closely related to infection-induced immunological abnormalities.

WGCNA differs from traditional DEG-based screening methods. It overcomes the disadvantages of traditional methods that only allow the partial analysis of datasets, thus possibly missing key regulatory molecules and making it challenging to explore and study the entire biological system. The network of individual biological interactions has been systematically mapped, and core prognosis-associated molecules have been identified [26, 27]. In this study, genes strongly linked to KD were found using WGCNA. The results of this study were then compared with those of another study that examined DEG to determine whether there were significant differences or correlations between the two sets of genes. Subsequently, six hub genes were identified through LASSO analysis: *FCGR1B*, *GPR84*, *HP*, *KREMEN1*, *LRG1*, and *TDRD9*. These six key genes were considerably overexpressed in the KD group compared to healthy controls.

*FCGR1* is an immunoglobulin G receptor with a high affinity that three gene families encode in humans [28]. One of the subtypes is designated by the acronym *FCGR1B*. *FCGR1B* was upregulated in patients with active tuberculosis and was associated with interferon (IFN)-stimulated gene expression, IL-1 production, and the NOD-like receptor signaling pathway [29]. *GPR84* is expressed by myeloid cells of the innate immune system, including neutrophils, monocytes, and macrophages. This receptor is a G protein-coupled medium-chain fatty acid receptor [30]. *GPR84* has been shown to play a unique role in innate immune cells and intestinal inflammation and is closely related to inflammasome activation [31]. HP is a plasma glycoprotein that binds to free hemoglobin and plays a crucial role in tissue protection and prevention of oxidative damage [32]. *KREMEN1* inhibits *Wnt* signaling [33]. It plays an important part in the control of cell death as well as the development of cancer [34, 35]. *LRG1* is an adipocytokine related to insulin resistance and angiogenesis [36, 37]. *TDRD9* is a member of the Tudor domain-containing protein family, primarily expressed in germ cells and closely related to azoospermia [38, 39]. *FCGR1B*, *GPR84*, *HP*, and *KREMEN1* are four of the six discovered hub genes associated with inflammation and the immune system. While *LRG1* is strongly associated with vascular homeostasis, the biological role of *TDRD9* in inflammation has not been previously identified and deserves further investigation.

These data support the notion that DEGs are associated with immune system reactions and inflammation. In many earlier investigations, KD has also been associated with monocytes, CD8^+^ T cells, and neutrophil infiltration [4, 5, 40]. This study is the first to use the ssGSEA methodology to investigate the infiltration by 28 different types of immune cells in KD tissues. KD tissue demonstrated considerably more infiltration of activated and effector memory CD4^+^ T cells, monocytes, neutrophils, and macrophages than healthy tissue. The results highlight that these cells are crucial to the course of KD. In addition, it was demonstrated that neutrophils stimulate DCs, and strong positive correlations were discovered between macrophages and the six hub genes. Six central and effector memory CD4^+^ T cells and central and effector memory CD8^+^ T cells were found to have an antagonistic interaction. Our results highlight the role of these immune cells and immunological regulatory genes in the development of KD [41, 42]. We utilized the MSigDB database of immunomarker genes as a reference for the GSEA of DEGs. This was done to investigate the likely mechanism by which immune function is maintained during the progression of KD. It was shown that neutrophils and peripheral blood mononuclear cells (PBMCs) have far more significant enrichment in DEGs. According to these studies, neutrophils and PBMCs are responsible for the onset and development of KD.

In this study, we screened for hub genes by analyzing the expression of differential genes in KD patients, and RT-qPCR further verified these results. The validated results indicated that the expression of *FCGR1B* (*P* < 0.001), *GPR84* (*P* < 0.001), *KREMEN1* (*P* < 0.001), *LRG1* (*P* < 0.001), and *TDRD9* (*P* < 0.001) was significantly upregulated in KD patients compared with the control group, while *HP* (*P* > 0.05) was not significantly upregulated in KD patients compared to the normal group (Figure 10). The results suggested that *FCGR1B*, *GPR84*, *KREMEN1*, *LRG1*, and *TDRD9* could be used as hub genes for screening KD. However, whether these hub genes are directly related to coronary artery damage in KD patients need further study.

In summary, using WGCNA and LASSO regression in conjunction with ssGSEA bioinformatic analysis, we found one hub module (blue module) and six hub genes (*FCGR1B*, *GPR84*, *HP*, *KREMEN1*, *LRG1*, and *TDRD9*) that may be associated with the development of KD. Five of the six hub genes (*FCGR1B*, *GPR84*, *KREMEN1*, *LRG1*, and *TDRD9*) were validated. This study gives early information on KD's immune infiltration pattern and immunomodulatory mechanisms. Prospective and large-sample follow-up studies should detect the diagnostic indicators of KD described here with high sensitivity and specificity, minimize misdiagnosis of KD, and serve as a guide for the early diagnosis and successful pharmacological treatment of KD. The limitations of this study are as follows: first, as the study mainly relied on published RNA microarray datasets for analysis, key clinical data could not be obtained. Second, the mechanism of action of these identified hub genes needs to be further studied.

## Figures and Tables

**Table 1:** Top 15 significant immunological signatures enriched by DEGs in GSEA.

Gene set name	NES	*P*-value	NOM p.adjust	FDR *q*-values
GSE22886 NAÏVE BCELL VS. NEUTROPHIL DN	3.221629	1.00E-10	2.93E-09	1.83E-09
GSE6269 HEALTHY VS. STAPH PNEUMO INF PBMC DN	3.19896	1.00E-10	2.93E-09	1.83E-09
GSE7400 CTRL VS. CSF3 IN VIVO TREATED PBMC DN	3.180609	1.00E-10	2.93E-09	1.83E-09
GSE6269 E COLI VS. STREP PNEUMO INF PBMC DN	3.135927	1.00E-10	2.93E-09	1.83E-09
GSE6269 FLU VS. STAPH AUREUS INF PBMC DN	3.102818	1.00E-10	2.93E-09	1.83E-09
GSE22886 NAÏVE TCELL VS. NEUTROPHIL DN	3.097563	1.00E-10	2.93E-09	1.83E-09
GSE4748 CYANOBACTERIUM LPSLIKE VS. LPS AND CYANOBACTERIUM LPSLIKE STIM DC 3 H DN	3.078676	1.00E-10	2.93E-09	1.83E-09
GSE29618 MONOCYTE VS. PDC UP	3.035813	1.00E-10	2.93E-09	1.83E-09
GSE22886 NAÏVE TCELL VS. MONOCYTE DN	3.030273	1.00E-10	2.93E-09	1.83E-09
GSE22886 NAÏVE CD4^+^ TCELL VS. MONOCYTE DN	3.014044	1.00E-10	2.93E-09	1.83E-09
GSE4984 UNTREATED VS. GALECTIN1 TREATED DC UP	2.999805	1.00E-10	2.93E-09	1.83E-09
GSE29618 MONOCYTE VS. PDC DAY7 FLU VACCINE UP	2.998979	1.00E-10	2.93E-09	1.83E-09
GSE34205 HEALTHY VS. RSV INF INFANT PBMC DN	2.991961	1.00E-10	2.93E-09	1.83E-09
GSE29618 MONOCYTE VS. MDC UP	2.991289	1.00E-10	2.93E-09	1.83E-09
GSE22886 NAÏVE CD8^+^ TCELL VS. MONOCYTE DN	2.976089	1.00E-10	2.93E-09	1.83E-09

DEGs, differentially expressed genes; GSEA, gene set enrichment analysis; NES, normalized enrichment score; NOM, nominal; FDR, false discovery rate.

## Data Availability

This research evaluated public databases. All the source data applied in this research came from the GEO data platform, which is open to the public (https://www.ncbi.nlm.nih.gov/geo/; accession numbers: GSE18606, GSE73461, and GSE68004).

## Abbreviations

KD: Kawasaki disease
WGCNA: Weighted gene coexpression network analysis
GEO: Gene Expression Omnibus
KEGG: Kyoto Encyclopedia of Genes and Genomes
GSEA: Gene set enrichment analysis
ssGSEA: Single sample of GSEA
DEGs: Differentially expressed genes
FCGR1: Fc gamma RI
GPR84: G protein-coupled medium-chain fatty acid receptor
Hp: Haptoglobin
KREMEN1: Kringle-containing transmembrane protein 1
LRG1: Leucine-rich *α*-2-glycoprotein 1
TDRD9: Tudor domain-containing protein 9
SVM-RFE: Support vector machine recursive feature elimination
LASSO: Least absolute shrinkage and selection operator
AUC: Area under the receiver operating characteristic curve
CALs: Coronary artery lesions
GO: Gene Ontology
MSigDB: Molecular Signatures Database.

## Supplementary Materials

Supplementary 1 The primers used for RT-qPCR with their sequence.

Supplementary 2 Weighted gene coexpression network analysis (WGCNA) establishes the effectiveness of the soft threshold. Analysis of the scale-free fit index and the average degree of connection for a range of soft-threshold powers (*ß*). The red line denotes the position of the point when the correlation coefficient equals to 0.85, and the soft-thresholding power equals to 3.

Supplementary 3 Weighted gene coexpression network analysis (WGCNA) establishes the effectiveness of the soft threshold. A histogram depicts the distribution of connections and verification of scale-free topology for *ß* = 3.

Supplementary 4 Dissolution curve of RT-qPCR experimental genes.
