# Supplementary material for: Identification of Hub Biomarkers and Immune and Inflammation Pathways Contributing to Kawasaki Disease Progression with RT-qPCR Verification
Source: J Immunol Res. 2023 Apr 6;2023:1774260. doi: 10.1155/2023/1774260 (PMC11637630; doi:10.1155/2023/1774260)
Supplement: Supplementary 1 — The primers used for RT-qPCR with their sequence. [file 1774260.f1.pdf]

TABLE S1. The primers used for RT-qPCR with their sequence

Gene	Forward primer (5' to 3')	Reverse primer (5' to 3')
FCGR1B	CTCCTTCTACATGGGCAGCAAGAC	GCAGCCTCGCACCAGTATAACC
GPR84	AGCCAATTAAAGGAGCCAGAAGAGC	TCCAGAATGTTGAGCAGCAAGAAGG
HP	AGGCATTATGAAGGCAGCACAGTC	CGCATCGCCATAGCAGGTGTC
KREMEN1	AGTCTATGGTCTGGCAACTCTCCTC	GAAGTCCCTGGTTGATGACAATCCC
LRG1	GATCTAACCCGAAACGCCCTGAC	GTAGCCACGAGACCTCCAGGAC
TDRD9	ATCAATGCGACTGGATCTACGATGC	GGTGCGAATAACATGCTGAGGAGAG
