# Supplementary figures and images for: Identification of Hub Biomarkers and Immune and Inflammation Pathways Contributing to Kawasaki Disease Progression with RT-qPCR Verification
Source: J Immunol Res. 2023 Apr 6;2023:1774260. doi: 10.1155/2023/1774260 (PMC11637630; doi:10.1155/2023/1774260)

### Scale independence

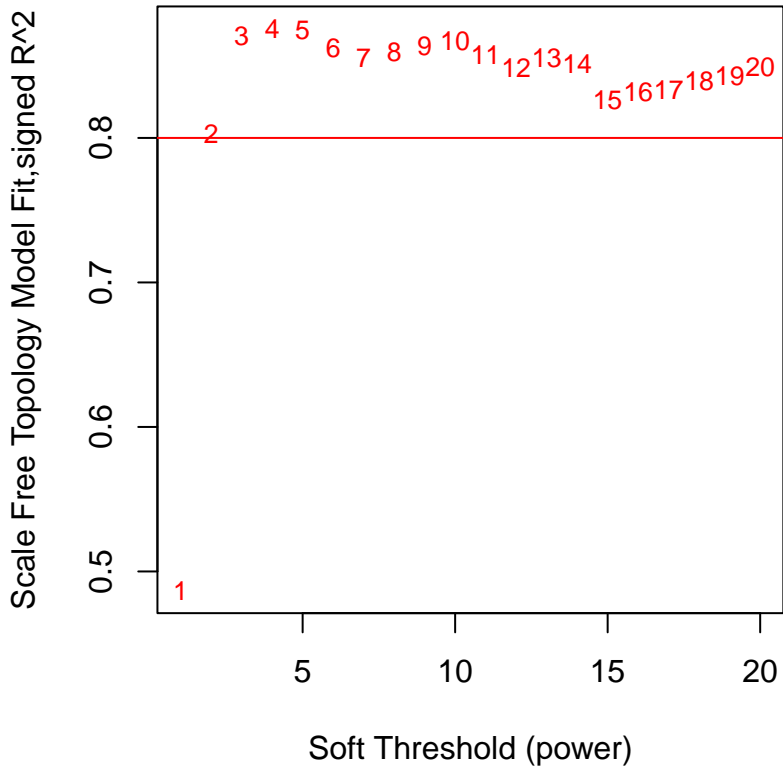

### Mean connectivity

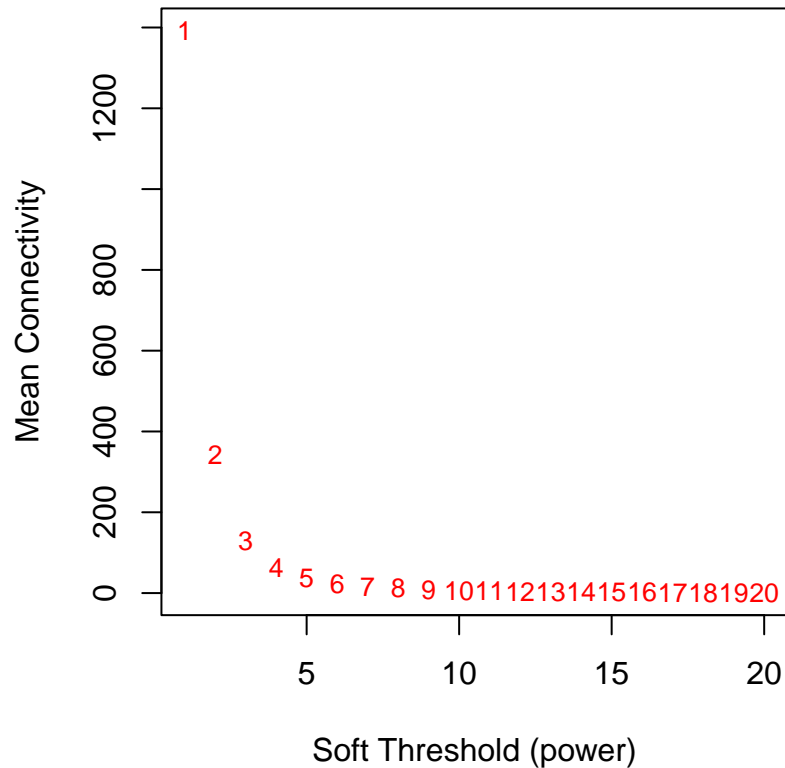

Supplement: Supplementary 2 — Weighted gene coexpression network analysis (WGCNA) establishes the effectiveness of the soft threshold. Analysis of the scale-free fit index and the average degree of connection for a range of soft-threshold powers (ß). The red line denotes the position of the point when the correlation coefficient equals to 0.85, and the soft-thresholding power equals to 3. [file 1774260.f2.pdf]

**Histogram of k**

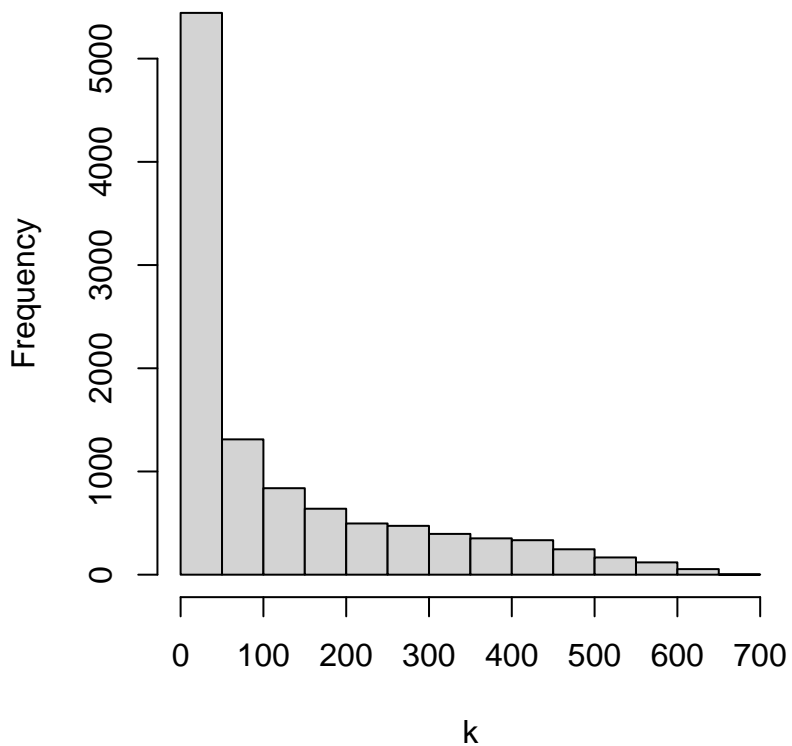

**Check Scale free topology**  
scale  $R^2 = 0.85$ , slope =  $-1.21$

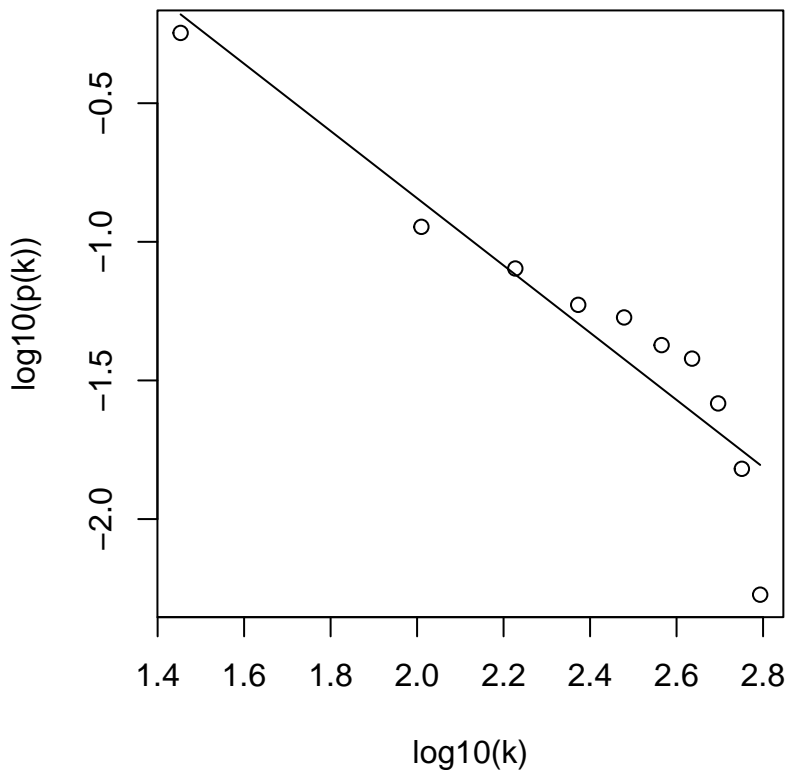

Supplement: Supplementary 3 — Weighted gene coexpression network analysis (WGCNA) establishes the effectiveness of the soft threshold. A histogram depicts the distribution of connections and verification of scale-free topology for ß = 3. [file 1774260.f3.pdf]

Dissolution Cure of RT-qPCR Experimental Genes

TDRD9

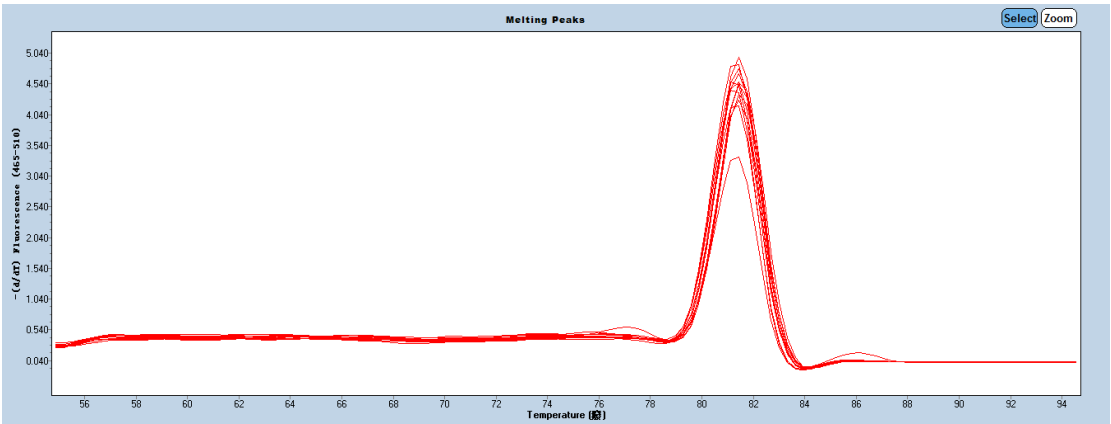

LRG1

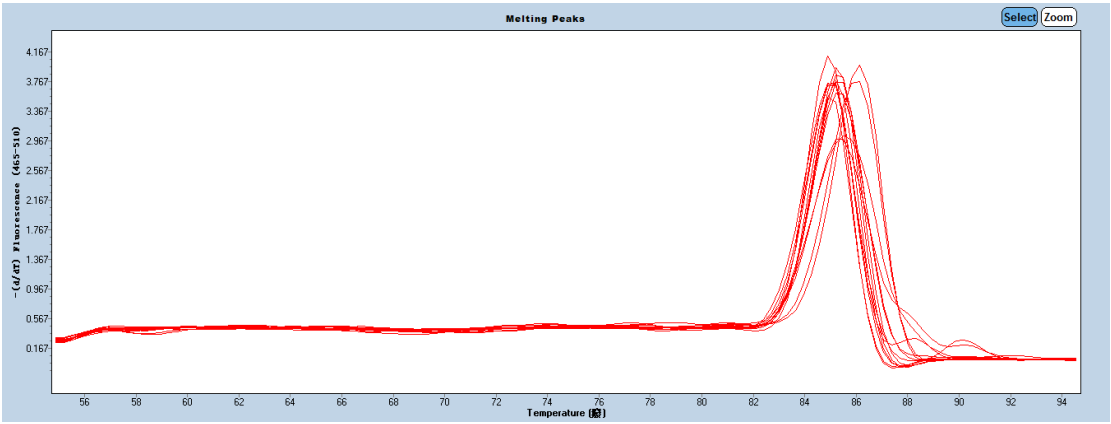

KREMEN1

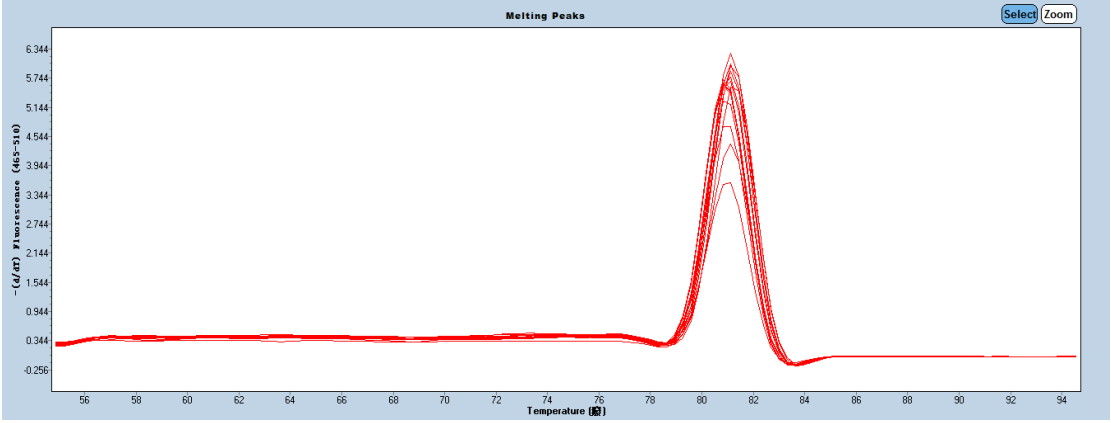

HP

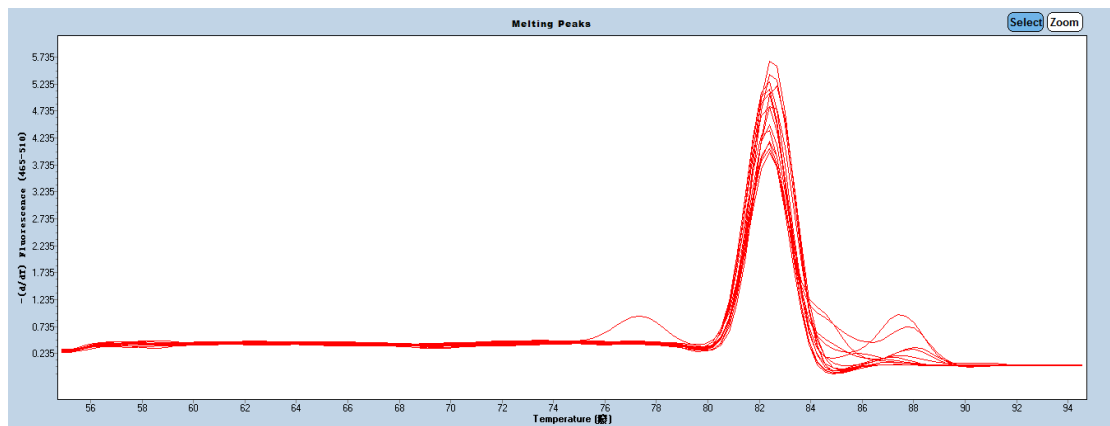

GPR84

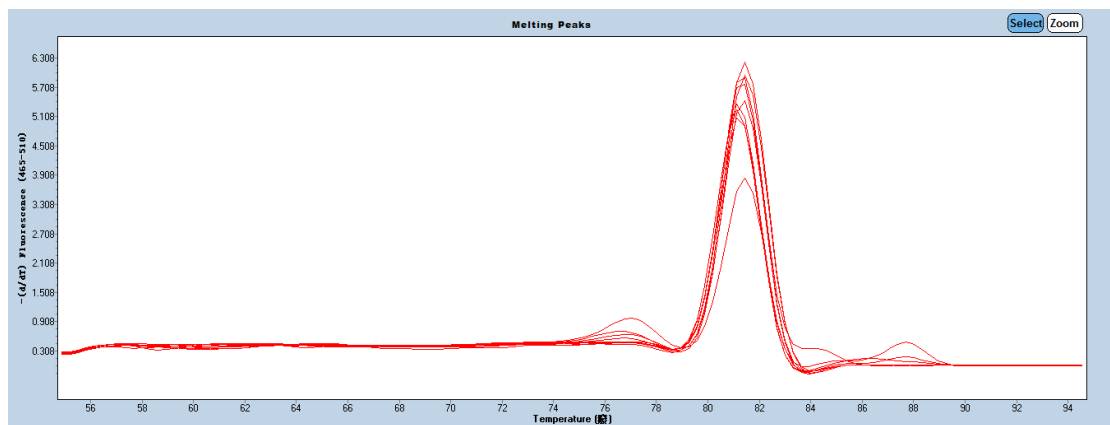

FCGR1B

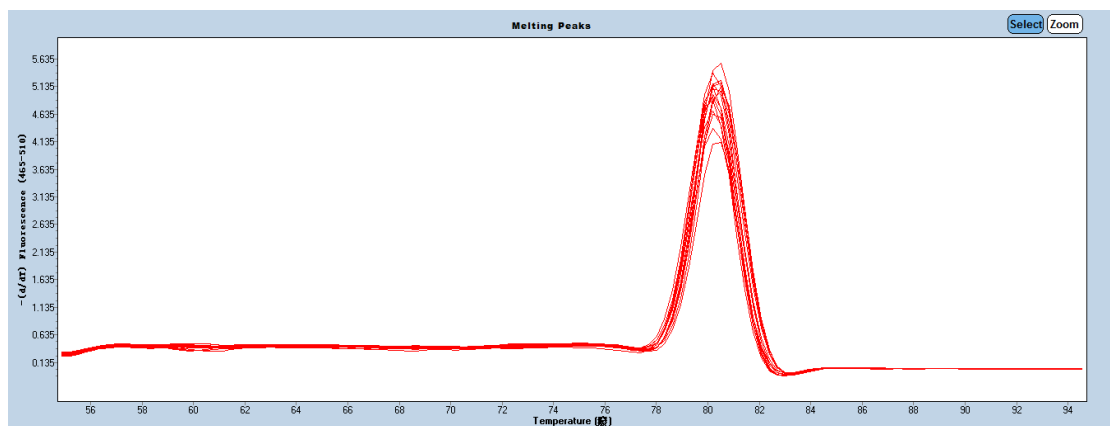

## GAPDH

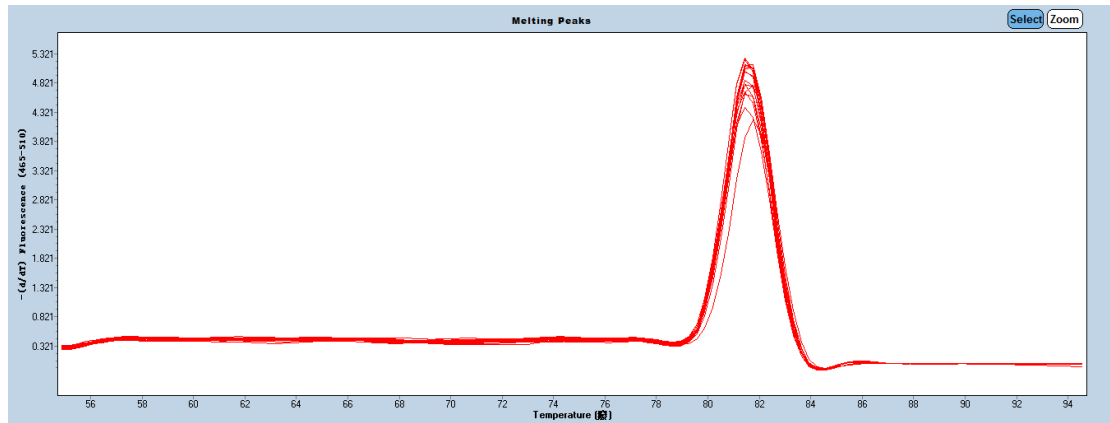

Supplement: Supplementary 4 — Dissolution curve of RT-qPCR experimental genes. [file 1774260.f4.pdf]
